# Repair cell first, then regenerate the tissues and organs

**DOI:** 10.1186/s40779-021-00297-5

**Published:** 2021-01-15

**Authors:** Xiao-Bing Fu

**Affiliations:** Wound Healing Unit, Medical Innovation Department and the Fourth Medical Center, PLA General Hospital and PLA Medical College, 28 Fu Xing Rd, Beijing, 100853 China

**Keywords:** Cell repair, Tissue repair, Regenerative medicine, Tissue engineering

## Abstract

Wound healing, tissue repair and regenerative medicine are in great demand, and great achievements in these fields have been made. The traditional strategy of tissue repair and regeneration has focused on the level of tissues and organs directly; however, the basic process of repair at the cell level is often neglected. Because the cell is the basic unit of organism structure and function; cell damage is caused first by ischemia or ischemia-reperfusion after severe trauma and injury. Then, damage to tissues and organs occurs with massive cell damage, apoptosis and even cell death. Thus, how to achieve the aim of perfect repair and regeneration? The basic process of tissue or organ repair and regeneration should involve repair of cells first, then tissues and organs. In this manuscript, it is my consideration about how to repair the cell first, then regenerate the tissues and organs.

## Main text

The cell is the basic unit of organism structure and function; cell damage is caused first by ischemia or ischemia-reperfusion after severe trauma and injury. Then, damage to tissues and organs occurs with massive cell damage, apoptosis and even cell death. Thus, the basic process of tissue or organ repair and regeneration should involve repair of cells first, then tissues and organs.

The traditional strategy of tissue repair and regeneration has focused on the level of tissue and organs directly; however, the basic process of repair at the cell level is often neglected. For example, ischemia- reperfusion is a basic pathological process of tissue injury caused by gunshot injury, trauma, burn and even diseases such as diabetic foot and venous ulcers. Histological findings show that three histological changes exist in skin wounds:1) necrotic tissue in the center of wounds, 2) apoptotic tissue area near the necrotic tissue and 3) ischemic-damaged tissue areas outside the apoptotic tissue area. Usually, ischemic-damaged tissues can recover their structure and function after debridement and infection control and are considered the tissues that will play a role in the repair process after debridement.

However, all methods currently used after debridement are directly for repair of tissue at the level of tissue and organs, without considering cell repair. For example, skin wound healing is accelerated with growth factor therapy. Typically, growth factors such as epidermal growth factor or fibroblast growth factor are used locally in the wound area after debridement [1, 2]. The current study identified that a combination of small molecules and growth factors could effectively induce IMR-90 fibroblasts into dopaminergic neuron-like cells that expressed dopaminergic neuron markers and possessed the electrophysiological properties of neurons [3]. The small molecule-based conversion approach may be potentially translated into therapeutic applications [4]. However, some cells in an apoptotic or ischemic condition in the wound area are stimulated by growth factors, and these damaged cells cannot play a normal role in repair. According to previous studies, the growth factors play a key role in tissue repair and regeneration after injury [5, 6], and their receptors on the membrane of these damaged cells are destroyed or their receptor function is down regulated, so they cannot have a role in function. The growth factors cannot play their maximum function in the damaged but not dead cells. The same is true for other methods used in tissue repair and regeneration, such as optics, electricity and magnetism. Therefore, the key way to achieve the best results in repair is to repair these damaged cells first, then tissue and organs. This is my personal view on tissue repair and regenerative medicine.

What can be used to repair the cells first? As mentioned before, ischemic cell injury is the basic pathological process of tissue injury caused by ischemia or ischemia-reperfusion after severe trauma and diseases. New management methods should focus on the key point of ischemia-reperfusion injury to reduce the injury by removing reactive oxygen species. Many methods or drugs have been used to scavenge active oxygen radicals, such as superoxide dismutase, vitamin C and the Saponins, a Chinese herbal medicine that has antioxidant effects. So a proper treatment procedure should include effective systemic resuscitation after severe injury first. Then, debridement by surgery or enzymes will help remove the necrotic tissue in the wound bed to reduce the colonization of bacteria and risk of infection and avoid further cell damage. Third, the microenvironment must be reestablished in the wound bed with local management, which may help the ischemic cells recover their function. Moisture wound dressings, such as alginate dressings, hydrocolloidal dressings and hydrogel dressings may help establish a moist wound-healing environment. A moist wound bed will benefit cell differentiation and proliferation to repair tissue and organs. Negative-pressure wound therapy is the best choice in local wound management to prepare the wound bed. Some antioxidant drugs could be used in the local wound bed or in veins to reduce ischemic cell damage and help cells recover their function after debridement. This is the first step to repair cells and accelerate the perfect tissue repair and regeneration at the level of tissues and organs. After then, the microenvironment recovery in the wound bed with debridement and use of advanced dressings will benefit cell repair.

Dedifferentiation of cells in situ in tissues plays a key role in tissue repair and regeneration. Recently, cell dedifferentiation in vitro or in vivo and its role in tissue repair and regeneration have been highlighted. Such as, the neuroregeneration and plasticity [7] and bone defect repair [8], have been an area of concern because of high incidence and lack of clear and effective treatment strategies. The results will help in understanding the mechanisms and process of perfect repair and regeneration at the cell level.

## Conclusions

The active cells with normal function are the basis of perfect tissue repair and regeneration. The window for repair cell first in all processes of tissue repair and regeneration include effective systemic resuscitation of severe trauma to alleviate ischemia-reperfusion injury in the early stage, then to scavenge active oxygen radicals in the wound bed immediately after debridement, and finally to maintain a good wound microenvironment with modern dressings and local application of antioxidant drugs to further reduce injury caused by active oxygen radicals during the whole treatment process.

## Data Availability

Not applicable.
